# Agile Robotic Fliers: A Morphing-Based Approach

**DOI:** 10.1089/soro.2017.0120

**Published:** 2018-10-10

**Authors:** Valentin Riviere, Augustin Manecy, Stéphane Viollet

**Affiliations:** ^1^ Aix Marseille University, CNRS, ISM, Marseille, France.; ^2^ ONERA Midi-Pyrenees Toulouse Center, Toulouse, France.

**Keywords:** morphing robot, quadrotors, obstacle avoidance, bioinspired, aerial robot, elastic folding mechanism

## Abstract

The aerial robot presented here for the first time was based on a quadrotor structure, which is capable of unique morphing performances based on an actuated elastic mechanism. Like birds, which are able to negotiate narrow apertures despite their relatively large wingspan, our Quad-Morphing robot was able to pass through a narrow gap at a high forward speed of 2.5 *m.s*^− 1^ by swiftly folding up the structure supporting its propellers. A control strategy was developed to deal with the loss of controllability on the roll axis resulting from the folding process, while keeping the robot stable until it has crossed the gap. In addition, a complete recovery procedure was also implemented to stabilize the robot after the unfolding process. A new metric was also used to quantify the gain in terms of the gap-crossing ability in comparison with that observed with classical quadrotors with rigid bodies. The performances of these morphing robots are presented, and experiments performed with a real flying robot passing through a small aperture by reducing its wingspan by 48% are described and discussed.

## Objective

Flying through cluttered environments requires an outstanding level of agility, which often involves the ability to trigger aggressive maneuvers to quickly avoid obstacles or pass through gaps at high speed. In the living world, agility is not restricted to flying insects or even small birds such as hummingbirds. Larger birds such as goshawks^1^ and budgerigars^2^ are able to negotiate cluttered environments at high speed despite their relatively large wingspan. How do they manage to perform tasks of this kind? By morphing their shape dynamically and reducing their wingspan swiftly by tucking up their wings. Morphing abilities give a flying robot agility by momentarily reducing its wingspan while keeping a sufficiently high payload. Morphing does not require any aggressive maneuvers but fast embedded mechanisms for folding up the robot's structure, as described in Ref.^3^ for a winged drone.

In the field of robotics, unmanned aerial vehicles are being used increasingly in cluttered and indoor environments for various purposes such as search and rescue expeditions,^4^ mapping,^5^ and exploration.^6^ The latest flying robots, therefore, have to be able to avoid collisions and handle narrow gaps successfully. Quadrotors, with their hovering and vertical takeoff and landing abilities, are certainly among the best candidates for meeting these requirements.

Here we focused on designing a narrow gap-crossing strategy that was implemented on a quadrotor. One previous strategy, which has been widely studied, consisted in performing aggressive maneuvers to make the quadrotor change its attitude swiftly to pass through a vertical or tilted window.^7^ Recent studies Refs.^8,9^ have succeeded in developing autonomous robotic gap-crossing skills based on on-board sensing and computing processes. However, this aggressive attitude control approach has several limitations: the robots have to reach high velocities and angular accelerations that require low inertia of the robot's body as well as high sensor refresh rates, especially in the case of visual sensors so as to prevent blur motion and maintain accurate position estimation with respect to the gap to be crossed.

To address this issue, a new approach was adopted based on morphological changes. Previous authors have presented various types of quadrotors in which the size of the structure can be adapted either passively or dynamically for different purposes. Nonactuated structures were used by Ref.^10^ to ensure resilience to collision and by Ref. ^11^ to obtain a self-deployable system facilitating the robot's transport. Actuated structures were used by Ref.^12^ to reduce the wingspan of a hovering robot or to reduce the robot's volume with a scissor-like foldable structure in Ref.^13^ Simulated robotic platforms with morphing abilities have been endowed by Ref.^14^ with full attitude control and by Ref.^15^ with an interesting tilting rotor mechanism.

Here we present a novel morphing approach whereby a flying quadrotor is endowed with an actuated elastic morphing structure that enables it to cross any gaps encountered at high speed. As shown in the supplementary video, the folded robot is able to pass through apertures that are narrower than the unfolded robot. Material and Methods section describes in detail the design and the dynamic model on which the morphing robot's structure was based. The control laws and strategy used to stabilize the robot during the folding and unfolding steps are described in the Gap-crossing scenario section. The experimental results presented in the Results section show the performances of which the Quad-Morphing robot is capable.

## Material and Methods

### Hardware and software overview

**FIG. 1.**
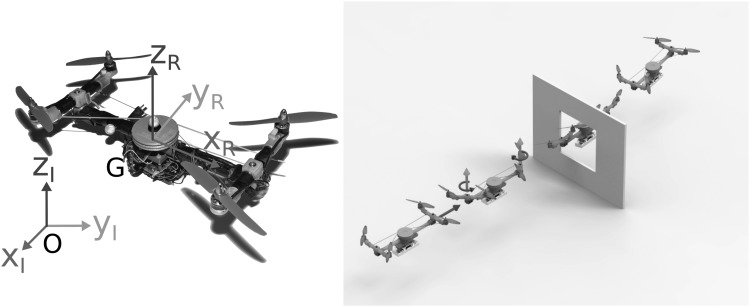
*Left:* Photo of the Quad-Morphing platform. *Right:* Computer-aided-design view of the Quad-Morphing platform flying toward a gap while rotating (folding) the arms supporting its four propellers to reduce its wingspan smoothly and quickly so as to avoid colliding with the gap.

**FIG. 2.**
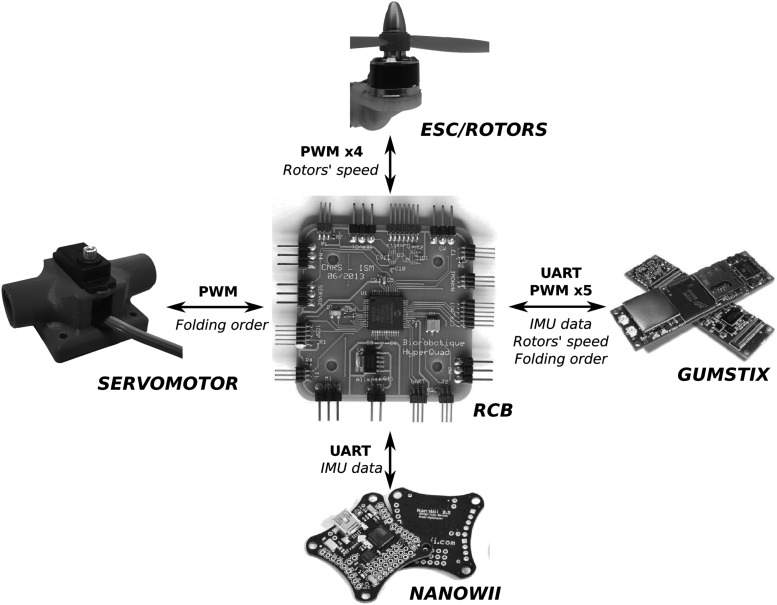
Hardware description: all the signals are transmitted through the custom-made RCB giving a feedback control of the propeller speeds and voltage levels. RCB, rotor controller board.

As shown in Figure 1, we designed and constructed an aerial robotic platform (Quad-Morphing robot) to test the ability of an aerial robot to pass through a gap smaller than its wingspan without any need of aggressive maneuvers. The hardware architecture of the Quad-Morphing robot was based on a previous custom-made platform developed at our laboratory,^16^ which consisted of the following (Fig. 2):

A Gumstix^®^ Overo^®^'s linux-based computer-on-module for high-level control (attitude/position/folding control) and communication with the ground station through WiFi.
A NanoWii board carrying a six-axes inertial measurement unit (IMU MPU6050), which can be used to control manually the quadrotor.
A very fast and accurate servomotor (MKS DS92A+) actuating the folding system.
A rotor controller board: a custom-built board giving a low-level control of the rotational speed propellers and providing connections between all the components.

The servomotor implemented on the robot was as fast and light as possible. The folding system was designed so that the two arms could rotate concomitantly to fold or unfold the structure, using a single actuator. Further details about the folding system are given in the following section.

### Mechanical design and model

**FIG. 3.**
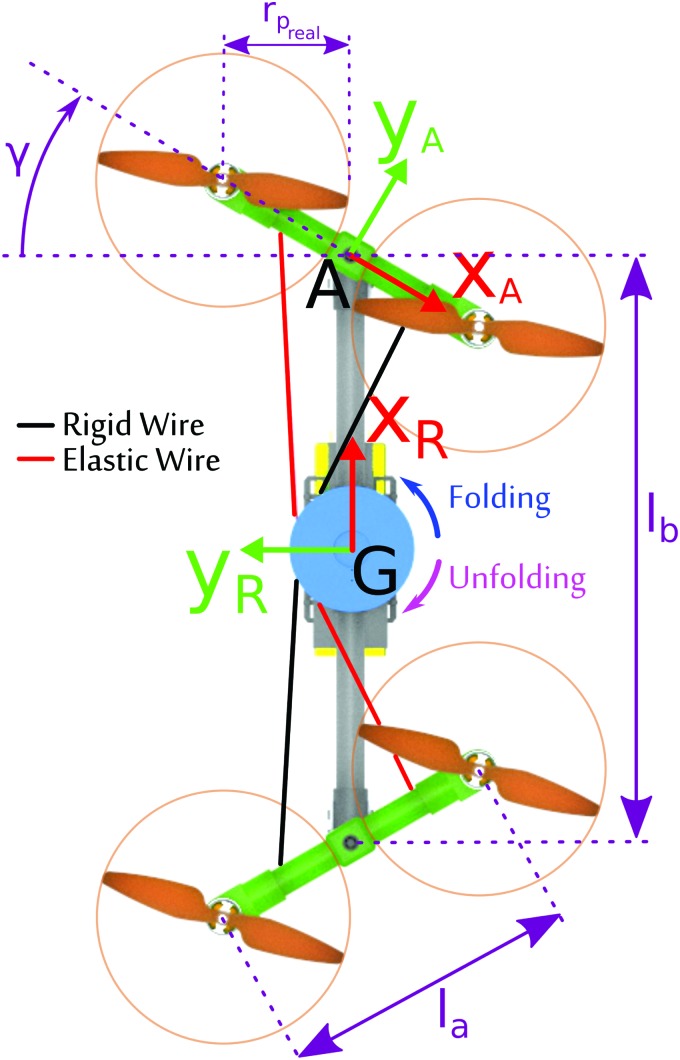
Linkage scheme for the Quad-Morphing robotic platform equipped with an elastic folding mechanism. A servomotor drives the pulley to make the two arms carrying the propellers rotate quickly, resulting in the folding (or unfolding) of the robot's structure. The angle $\gamma$, called the folding angle, can range between 0° (unfolded) and 90° (folded). Color images available online at www.liebertpub.com/soro

As shown in Figure 3, the Quad-Morphing robot was based on a classical quadrotor platform, the mechanical structure of which was greatly adapted to make the robot able to reduce its wingspan dynamically. To simplify the mechanical design, a straightforward approach was used, which consisted in using two pivot links to allow the two arms supporting the robot's propellers to rotate. To keep the robotic platform as light as possible, we implemented a wire-based mechanism composed of two rigid wires fixed to a rotating pulley (blue in Fig. 3) mounted onto a fast servomotor and one elastic wire fixed to the edges of the arms and held taut by means of the pulley groove. The tension of the elastic wire was adjusted so as to facilitate the smooth folding and unfolding of the structure and to keep the structure rigid whatever the arms' positions, with no backlash. This mechanism enables the flying robot to fold its arms dynamically back against its body (Fig. 3: $\gamma \in { [ 0 , 90^ \circ} ]$) and thus to greatly reduce its wingspan to the diameter of a single propeller. Between the two extreme positions: folded $( \gamma { = 90^ \circ} )$ and unfolded $( \gamma { = 0^ \circ } )$, the robot can reach every intermediate position with a precision that depends on the angular precision of the servomotor (almost 2°).

#### Size parameters

The Quad-Morphing robot was designed with a 400 g payload. The body length *l_b_* and arm length *l_a_* (Fig. 3) were sized with respect to the propeller's radius ${r_{{p_{real}}}}$. During the design phase, we also examined the use of a slightly larger propeller radius (${r_p} = 70 \,mm$) than the actual length (${r_{{p_{real}}}} = 64 \,mm$) to prevent the propellers from touching each other. The robot had the following main dimensions, as shown in Figure 3:

\begin{align*}
\begin{matrix} {{r_{{p_{_{real}}}}}} \hfill & {  = 64 \,mm} \hfill \\ {{r_p}} \hfill & {  = 70 \,mm} \hfill \\ {{l_a}} \hfill & {  = 2.{r_p} = 140 \,mm} \hfill \\ {{l_b}} \hfill & {  = 4.{r_p} = 280 \,mm} \hfill \\ \end{matrix} \tag{1}
\end{align*}

#### Dynamic model

To rotate from the inertial frame $\mathcal{I}$ to the robot's frame $\mathcal{R}$ (Fig. 1), we used ZYX Euler angles, which correspond to the rotation matrix ***R*** defined as follows:

\begin{align*}
\begin{matrix} { \psi: \,{ \rm{Yaw}} \,{ \rm{around}} \,{ \rm{the}} \,{ \rm{Z {\hbox{-}} axis}}} \hfill \\ { \theta: \,{ \rm{Pitch}} \,{ \rm{around \ the}} \,{ \rm{Y^{\prime} {\hbox{-}} axis}}} \hfill \\ { \phi: \,{ \rm{Roll}} \,{ \rm{around}} \,{ \rm{the}} \,{ \rm{X^{\prime \prime} {\hbox{-}} axis}}} \hfill \\ \end{matrix}
\end{align*}

In the first step, we modeled the Quad-Morphing robot using Newton's equations of motion for dynamic rigid bodies,^17^ including the thrust and torques applied to the robot, the gyroscopic effects, and the fluid resistance denoted by the coefficient *K_v_*:

**Figure:**
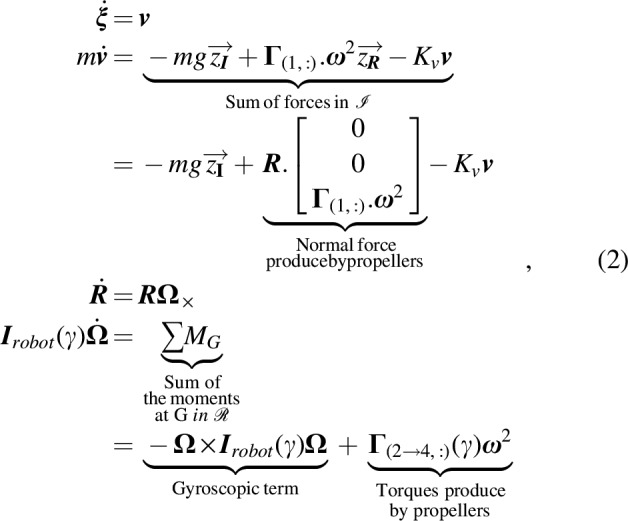


where ${\dot{{\upxi}}} = \textbf{\textit{v}}$ and $\textbf{\textit{\underline{v}}}$ are, respectively, the robot's speed and its acceleration expressed in the inertial frame $\mathcal{I}$, ${\bm{\Omega} _ \times }$ is the skew symmetric matrix of the body's rates, *m* is the mass, ${\textbf{\textit{I}}_{robot}}$ is the inertia matrix, $\textbf{\textit{R}}$ is the rotation matrix, $\Gamma$ is the control matrix [Eq. (5)], where ${\bm{\Gamma}_{ ( 1 ,: ) }}$ corresponds to the first line of the matrix $\Gamma$ and $\omega$ is the column vector of the propellers' rotational speed. The changes in the inertia ***İ***_*robot*_ were neglected since they were taken to be temporary disturbances occurring only during folding maneuvers, that is, during a very short period (about 200 ms, see Modeling the folding system section). The inertial accelerations of the arms canceled each other out because they moved in opposite directions during the folding and unfolding processes (Fig. 3).

Torques and thrust generated by the quadrotor depend on the square of the propellers' rotational speed, according to the propeller's physics^18^ as follows:

\begin{align*}
\begin{matrix} {{F_{{p_i}}} = {c_T}.{ \omega ^2}} \\ {{ \tau _{{p_i}}} = {c_D}.{ \omega ^2}} \\ \end{matrix} , \tag{3}
\end{align*}

where ${F_{{p_i}}}$ and ${ \tau _{{p_i}}}$ are the thrust force and drag moment produced by the *i*-propeller, respectively. *c_T_* and *c_D_* are the thrust and drag coefficients, respectively.

Torques on the roll and pitch axes also depend on the folding angle $\gamma$ (Fig. 3). The control matrix $\Gamma$ transforms each propeller's speed into a thrust force ${T_ \Sigma }$ and moments ${ \tau _{roll , pitch , yaw}}$ as follows:

\begin{align*}
\left[ { \begin{matrix} {{T_ \Sigma }} \\ {{ \tau
_{roll}}} \\ {{ \tau _{pitch}}} \\ {{ \tau _{yaw}}} \\
\end{matrix} } \right] = \left[ { \begin{matrix} { \mathop \sum
\limits_{i = 1}^4 {F_{{p_i}}}} \\ { \mathop \sum \limits_{i = 1}^4
{y_i}.{F_{{p_i}}}} \\ { -  \mathop \sum \limits_{i = 1}^4
{x_i}.{F_{{p_i}}}} \\ { \mathop \sum \limits_{i = 1}^4 { \tau
_{{p_i}}}} \\ \end{matrix} } \right] = \bm{\Gamma} ( \gamma ).
\underbrace{\left[ { \begin{matrix} { \omega _1^2} \\ { \omega
_2^2} \\ { \omega _3^2} \\ { \omega _4^2} \end{matrix} } \right]
}_{{ \omega ^2}} , \tag{4}
\end{align*}

where ${T_ \Sigma }$ and ${ \tau _{roll , pitch , yaw}}$ are the total thrust and torques applied to the three axes in the robot's frame, respectively. *x_i_* and *y_i_* are the distances on the X-axis and Y-axis of the *i*-propeller. The coefficients of the matrix $\bm{\Gamma}$ depend on geometrical parameters that are given by

**Figure:**
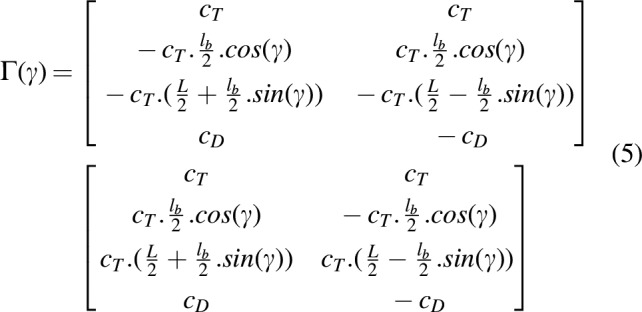


The coefficients of the matrix $\Gamma$ are updated in real time to take into account the changes occurring in the folding angle $\gamma$ due to the rotation of the servomotor (Fig. 3). As expected, in view of the design of the folding structure, the moment around the roll axis becomes uncontrollable when the folding angle is equal to 90° [see second line of matrix $\bm{\Gamma}$ in Eq. (5)].

#### Inertia

The robot's inertia $_ \mathcal{R}{\textbf{\textit{I}}_{robot}} ( G )$ during the folding process, calculated on the basis of the center of mass *G* with respect to the robot's frame $\mathcal{R}$, depends on the folding angle $\gamma$ and can be decomposed into the following three matrices: the body's inertia $_ \mathcal{R}{\textbf{\textit{I}}_{body}} ( G )$ and the two arms' inertia $_ \mathcal{R}{\textbf{\textit{I}}_{arm{_1}}} ( A )$ and $_ \mathcal{R}{\textbf{\textit{I}}_{arm{_2}}} ( A \prime )$, calculated on the basis of the arms' centers of mass *A* and *A*′, respectively.

\begin{align*}
_\mathcal{R}{\textbf{\textit{I}}_{robot}} ( G{ ) = _ \mathcal{R}}{\textbf{\textit{I}}_{body}} ( G ) { + _ \mathcal{R}}{\textbf{\textit{I}}_{arm{_1}}} ( A ) { + _ \mathcal{R}}{\textbf{\textit{I}}_{arm{_2}}} ( A \prime ) \tag{6}
\end{align*}

Computer-aided design software provided the inertia matrix terms in the inertial frame for the body and arms as follows:

\begin{align*}
\begin{split}&  _ \mathcal{R}{I_{body}} ( G ) = \left[ {
\begin{matrix} {{I_{{b_x}}}} & 0 & 0 \\ 0 & {{I_{{b_y}}}} & 0 \\ 0
& 0 & {{I_{{b_z}}}} \\ \end{matrix} } \right] \\ & _{ \cal
A}{I_{ar{m_1}}} ( A ) { = _{ \cal A}}{I_{ar{m_2}}} ( A^\prime ) =
\left[ { \begin{matrix} {{I_{{a_x}}}} & 0 & 0 \\ 0 & {{I_{{a_y}}}}
& 0 \\ 0 & 0 & {{I_{{a_z}}}} \\ \end{matrix} } \right] \end{split}
 \tag{7}
\end{align*}

The formula is calculated for just one arm by obtaining the arm's inertia on the robot's center of mass G in the robot's frame $\mathcal{R}$, using Steiner's theorem and the parallel axis theorem:

\begin{align*}
\begin{matrix} {_ \mathcal{R}{\textbf{\textit{I}}_{arm1}} ( A ) =
{\textbf{\textit{R}}_{{ \cal A} \to \mathcal{R}}}{._{ \cal
A}}{\textbf{\textit{I}}_{arm1}} ( A
).{\textbf{\textit{R}}^\textbf{\textit{t}}}_{{ \cal A} \to
\mathcal{R}}} & {} \\ {{ \rm{with}} \,{\textbf{\textit{R}}_{{ \cal
A} \to \mathcal{R}}} = \left[ { \begin{matrix} { \cos ( \gamma ) }
& { \sin ( \gamma ) } & 0 \\ { -  \sin ( \gamma ) } & { \cos (
\gamma ) } & 0 \\ 0 & 0 & 1 \\ \end{matrix} } \right]
}.\end{matrix} \tag{8}
\end{align*}

**Figure:**
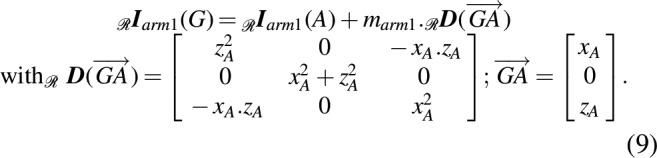


Substituting Equation (9) into Equation (6), we obtain

\begin{align*}
_\mathcal{R}{\textbf{\textit{I}}_{robot}} ( G ) = diag \left( { \begin{matrix} {{I_{bod{y_x}}} + 2. ( {I_{{a_x}}}. \mathop { \cos } \nolimits^2 ( \gamma ) + {I_{{a_y}}}. \mathop { \sin } \nolimits^2 ( \gamma ) ) + 2.{m_{arm}}.z_A^2} \\ {{I_{bod{y_y}}} + 2. ( {I_{{a_x}}}. \mathop { \sin } \nolimits^2 ( \gamma ) + {I_{{a_y}}}. \mathop { \cos } \nolimits^2 ( \gamma ) ) + 2.{m_{arm}}. ( x_A^2 + z_A^2 ) } \\ {{I_{bod{y_z}}} + {I_{{a_z}}} + 2.{m_{arm}}.x_A^2} \\ \end{matrix} } \right). \tag{10}
\end{align*}

As we can see, the inertial matrix $_ \mathcal{R}{\textbf{\textit{I}}_{robot}} ( G )$ stays diagonal during the folding process, which would not be the case if the arms were rotating in the same direction. We, therefore, chose the mechanical configuration described in Figure 3 with the two arms rotating in opposite directions. It can also be seen from Equation (10) that the inertia depends on the folding angle $\gamma$ on the roll and pitch axes. The controller's gain is, therefore, automatically adjusted, depending on the state of the robot's structure (folded or unfolded).

From Equation (10), the changes in the inertia occurring on each rotational axis due to the folding were calculated:

\begin{align*}
{\Delta {I_{robot_x}}} & = - 43 \%  \\  { \Delta {I_{robo{t_y}}}}
& = + 11 \%   \\ { \Delta {I_{robo{t_z}}}}
 & = 0 \%  . \tag{11} \end{align*}

As expected from the folding mechanism, the inertia on the roll axis decreases dramatically by 43%, whereas the inertia on the pitch axis increases by only 11%. The inertia on the yaw axis remains unchanged. The increase in the pitch inertia by about 11% of its initial value when the robot is unfolded led us to increase the proportional-integral-derivative (PID) controller gain on the pitch axis by this amount accordingly. The decrease in the roll inertia by about 43% reflects the existence of greater instability in the folded position, but the robot's stability was maintained passively by placing the center of mass below the center of thrust in our design.

### Modeling the folding system

**FIG. 4.**
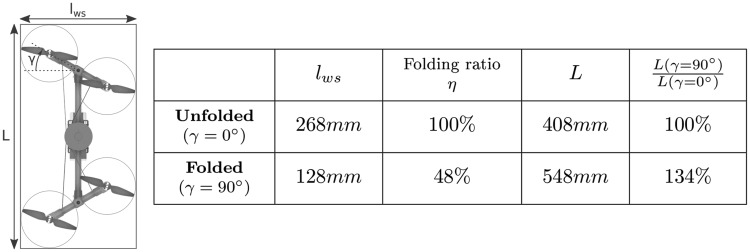
Wingspan and various length considerations about wingspan changes depending on the folding angle $\gamma$. The folding ratio [Eq. (13)] was defined to characterize the robot morphing ability in terms of wingspan reduction.

As shown in Figure 4, the servomotor regulates the robot's wingspan ${l_{ws}}$ by adjusting the folding angle $\gamma$, as well as by changing the robot's length *L* in line with the following equations (see Fig. 3 for definitions of the parameters):

\begin{align*}
\begin{split} {L ( \gamma ) } & { = {l_b} + 2.{r_{{p_{real}}}} +
{l_a}.sin ( \gamma ) } \\
{{l_{ws}} ( \gamma ) } & { = 2.{r_{{p_{real}}}} + {l_a}.cos (
\gamma ) } . \end{split}
 \tag{12}
\end{align*}

A new metric called the folding ratio, defined as the ratio between the folded and unfolded wingspan, is written as follows:

\begin{align*}
\eta = { \frac { { l_ { ws } } ( \gamma { { = 90 } ^ \circ } ) }  { { l_ { ws } } ( \gamma { { = 0 } ^ \circ } ) } } = { \frac { 2. { r_ { { p_ { real } } } } }  { 2. { r_ { { p_ { real } } } } + { l_a } } } . \tag { 13 } 
\end{align*}

As shown by Equation (13), the gain in terms of the wingspan is directly related to geometric parameters, namely the propeller radius and the length of the arms. In our prototype, the fully folded structure (i.e., $\gamma { = 90^ \circ }$) can adopt a wingspan ${l_{ws}}$ equal to 128 mm, which corresponds to a folding ratio of $\eta = 48 \%$, whereas the wingspan of the unfolded structure (i.e., $\gamma { = 0^ \circ}$) is equal to 268 mm (Fig. 4). In other words, the folded robot is more than two times smaller than the unfolded robot, which means that the robot can theoretically pass through a gap two times smaller than its usual wingspan. The same applies to the robot's length *L*: the folding ratio is also given in the case of the length in Figure 4. Once the wings have folded up, the increase in the robot body's length is 34%.

**FIG. 5.**
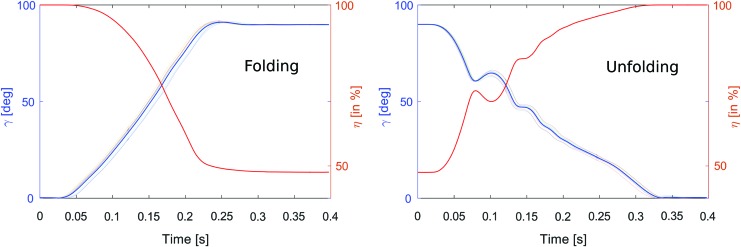
Folding angle (in *blue*) and folding ratio (in *red*) versus time. Color images available online at www.liebertpub.com/soro

Figure 5 shows experimental curves (eight distinct experiments are presented here) depicting the time course of the folding angle $\gamma$ and folding ratio $\eta$ during a folding and unfolding procedure. Data acquisition was made with a Vicon^®^ sytem motion capture. It can be noted in Figure 5 that small bumps appear only during unfolding due to the use of elastic wires in the mechanism (Fig. 3). However, the small amplitude of these fast bumps (lasting <50 ms) make them negligible.

The robot can achieve complete folding, that is, when the wingspan reaches 95% of its final value, within only $$\Delta {t_{fold}} = 230 \,ms$$ ($$mean = 230 \,ms , \,std = 6 \,ms , 8 experiments$$) and complete unfolding within only $$\Delta {t_{unfold}} = 310 \,ms$$ ($$mean = $$ $$ 310 \,ms , \,std = 5 \,ms , \,8 experiments$$).

### Gap-crossing scenario

**FIG. 6.**
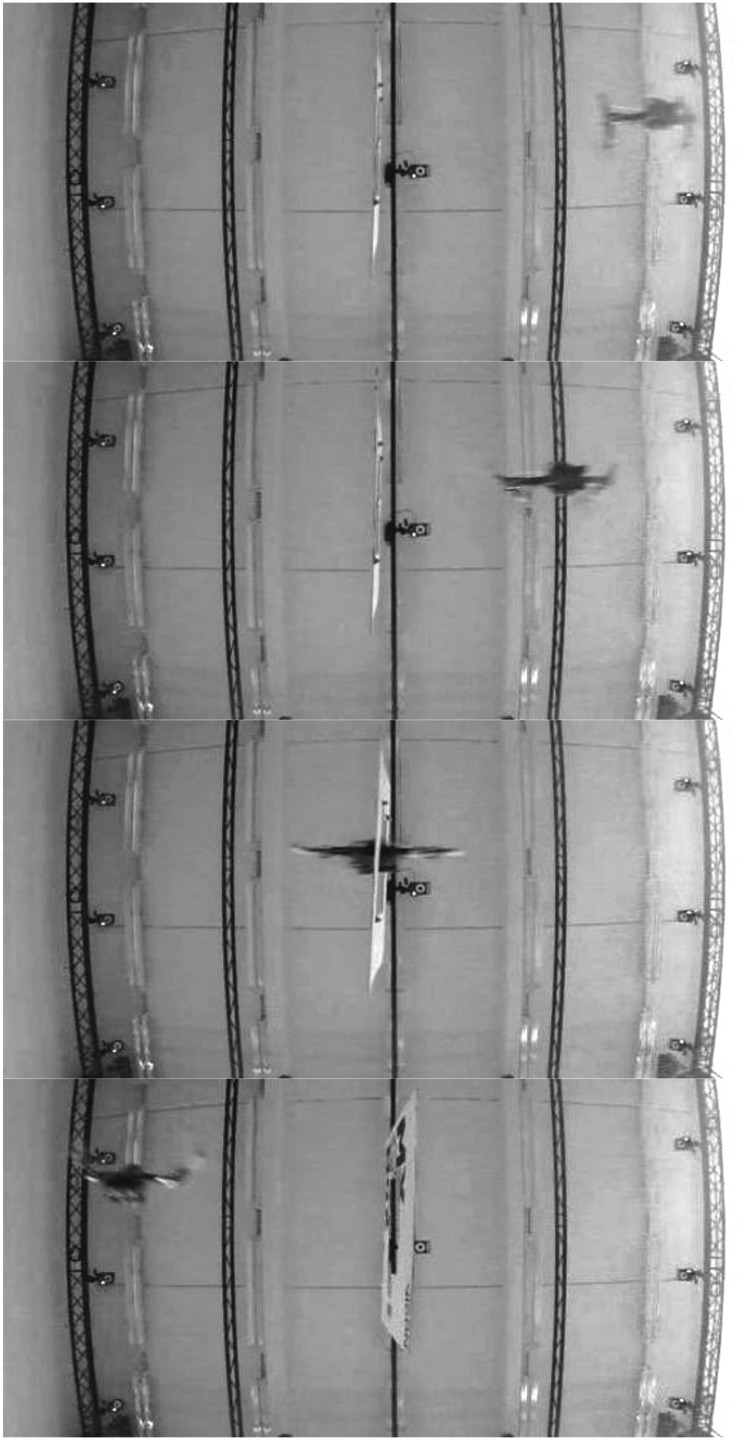
*Bottom view:* Sequence of the Quad-Morphing platform passing through a gap.

Here we present a scenario that consists in passing through a gap at high speed. The transversal crossing speed adopted on the X-axis was $2.5 \,m.{s^{ - 1}}$, which corresponds to a good trade-off between a short folding time $\Delta {t_{fold}}$ and the limited flight space available for our experiments. In all these experiments, the width of the gap was smaller than the unfolded robot's wingspan, which caused the robot to adopt the folded configuration, as shown in the frames extracted from a video-recorded gap-crossing test (Fig. 6).

**FIG. 7.**
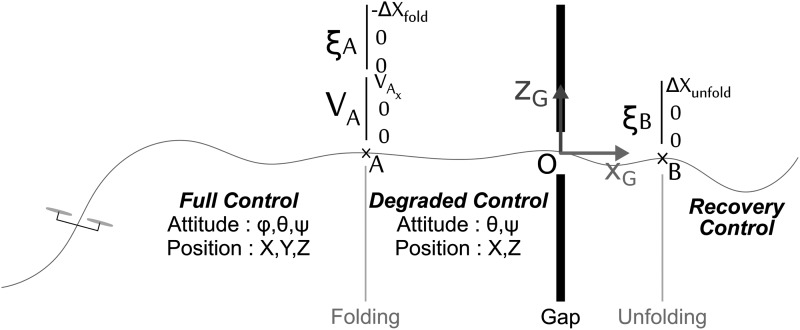
Side view of the gap-crossing scenario including the three modes that were activated sequentially, depending on the robot's position along its trajectory: (1) full control before reaching point A, (2) degraded control from points A to B, (3) recovery control after point B.

This scenario can involve three different modes, the activation of which depends on the position of the robot with respect to the gap (Fig. 7):

Full control mode: The attitude and position controllers are fully activated and run in real time onboard the robot. Thanks to the trajectory planner described in Figure 9, we can generate a feasible trajectory in terms of the acceleration and the speed to make the robot reach the speed ${V_{{A_x}}}$ as fast as $2.5 \,m.{s^{ - 1}}$ in the steady state when crossing point A (Fig. 7).
Degraded control mode: immediately after folding of the two arms, the integrator output signal [Eq. (15)] delivered by the roll attitude controller is held constant. The position of point A before the gap is determined by applying the following equation: $\Delta {X_{fold}} = {V_{{A_x}}}. \Delta {t_{fold}}$, where ${V_{{A_x}}}$ and $\Delta {t_{fold}}$ are the robot's speed at point A and the folding time, respectively.
Recovery control mode: once the gap has been passed, a recovery mode procedure is initiated to restabilize the robot at point B, which is defined by a position with respect to the position of the gap: $\Delta { X_ { unfold } } = \frac { { L ( \gamma = 90^ \circ ) } }  { 2 }$.

We also implemented a supervisor that makes it possible to switch sequentially between the three modes already described so as to adjust the gains and activate the various controllers as required, depending on the robot's position along the trajectory imposed by the planner.

### Control and state estimation

**FIG. 8.**
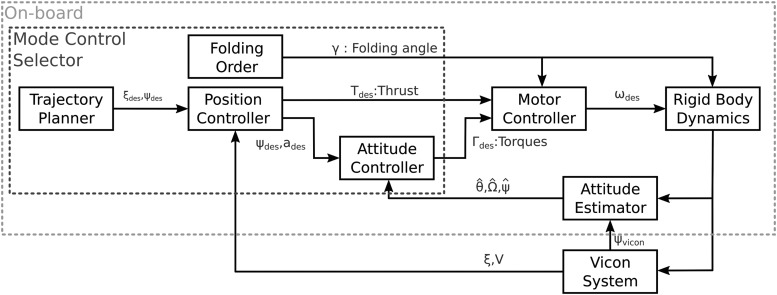
Block diagram of the Quad-Morphing robot's control system. *Gray dashed box* “On-board”: Components on-board (on the robotic platform); *black dashed box* “Mode Control Selector”: Components affected by the selected flight mode.

All the control laws, the estimator, and the trajectory planner described in Figure 8 are implemented on the Gumstix computer on module through the RTMaG toolbox^19^ developed at our laboratory and with QUARC^®^ running in the MATLAB^®^ environment in the ground station with WiFi communications. Position estimation and yaw measurements are provided by off-board sensors: a Vicon system of localization was used to determine the robot's position in real time and to emulate a magnetometer, which is not implemented on our platform. The motion capture system (the Vicon system) can locate with a great accuracy^16^ the Quad-Morphing robot in our flight arena, which is equipped with 17 cameras, with great accuracy.

Quadrotor robots are not fully actuated systems: four inputs corresponding to the four propeller's speeds control six outputs, which are the six degrees of freedom of the robot's rigid body (attitude and position in three dimensions). To control systems of this kind, we used two cascaded controllers (as described in Fig. 8): one position controller providing the thrust and two accelerations in the horizontal plane, and one attitude controller that receives horizontal accelerations and yaw angles as inputs and delivers outputs consisting of the three torques (roll, pitch, and yaw). The motor controller then determines the propellers' speeds by means of the control matrix $\bm{\Gamma}$, the inputs of which are the thrust and the three torques.

#### Position and yaw estimation

The 3D position and the heading are the only measurements acquired by the motion capture system at a sampling frequency of 500 Hz. The data are then filtered by a Kalman filter to reduce the noise and sent through WiFi at a frequency of 200 Hz to the Gumstix computer embedded on board.

#### Attitude estimation

A complementary filter^20^ was added for estimating the robot's attitude and debiasing the rate gyros. Unfortunately the IMU does not include any magnetometers for estimating the heading, which was specified in real time by the ground station through WiFi.

With the gyro and accelerometer measurements 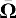 and 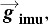 respectively, we obtain

**Figure:**
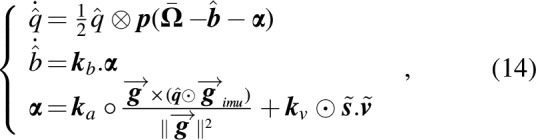


where 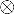 is the Hamilton product, 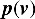 is defined by 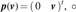 is the Hadamard product, and 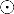 is the quaternion-vector product (defined by 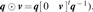). The quaternion error is defined by 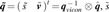 and ${{\tilde{v}}}$ are the scalar part and the vectorial part of the error, respectively, 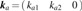 and 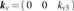 are weighting matrices that enable us to use the accelerometer alone to determine the roll and pitch values and the external Vicon to determine the yaw value.

#### Attitude and position controllers

The quaternion attitude controller implemented on-board based on Refs.^21,22^ ensures overall asymptotic stability. The following control law was adopted:

**Figure:**
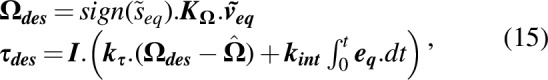


where 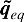 and 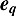 are the quaternion and the vector error, respectively, defined by

**Figure:**
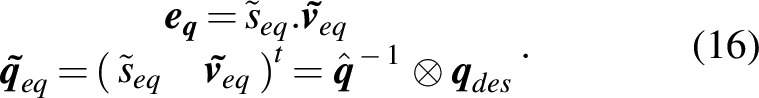


The attitude required is given directly by XY-axis accelerations and thrust adjustments imposed by the position controller:

\begin{align*}
\left( {\begin{matrix} { { \phi _ { des } } = \mathop { \sin }
\nolimits^ { - 1 } \left( { \frac { m }  { { { T_ { des } } } } (
{ a_ { 1 , des } } \sin \hat \psi - { a_ { 2 , des } } \cos \hat
\psi ) } \right) } \\ { { \theta _ { des } } = \mathop { \sin }
\nolimits^ { - 1 } \left( { \frac { m }  { { { T_ { des } } } } (
{ a_ { 1 , des } } \cos \hat \psi + { a_ { 2 , des } } \sin \hat
\psi ) } \right) } \\ \end{matrix}} \right.. \tag { 17 }
\end{align*}

The position controller implemented was a PID controller defined as follows:

**Figure:**
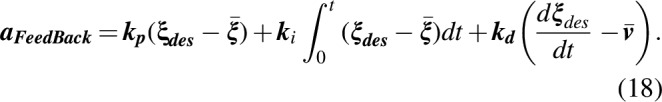


By adding a feed-forward term, the required thrust ${T_{des}}$ and acceleration ${a_{1:2 , des}}$ were directly obtained on the X and Y axes as follows:

\begin{align*}
\begin{cases} \begin{matrix} { { T_ { des } } = \frac { m }  {
{ \cos \hat \phi \cos \hat \theta } } \left( { { a_ { 3 , FeedBack
} } + g + { \frac { { d^2 } { \xi _ { 3 , des } } }  { d { t^2 } }
} } \right) } \hfill \\ { { a_ { 1:2 , des } } = { a_ { 1:2 ,
FeedBack } } + { \frac { { d^2 } { { \bm { \xi } } _ { 1:2 , des }
} }  { d { t^2 } } } } \hfill \\ \end{matrix} \end{cases} . \tag {
19 } \end{align*}

Numerical values of the controller coefficients are given by


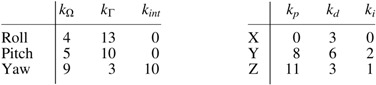


There is no direct adaptation term in the PID controller. The control adaptation is made by updating the coefficients of the control matrix $\Gamma$ as described in Mechanical design and model section.

#### Trajectory planning

**FIG. 9.**
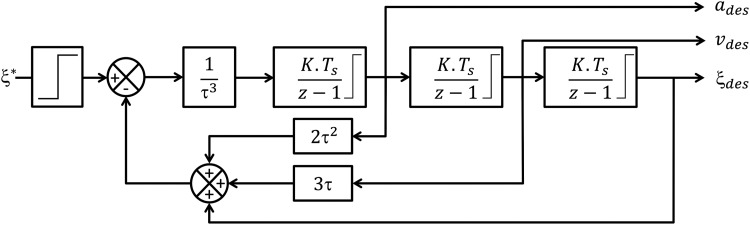
The trajectory planner generates accelerations, speeds, and positions compatible with feasible trajectory. The integrators are presented here in their discrete forward Euler form, where *T_s_* is the sampling time, which is equal to $10 \,ms$, $K = 1$, and $\tau =$ $500 \,ms$.

As described in Figure 9, a prefilter was added to generate speeds and accelerations that are compatible with the robot's dynamics. This trajectory planner consists of three cascaded integrators ensuring smooth accelerations, which are injected into the feed-forward position controller. Physical constraints (speed and acceleration limits) were also added to each integrator by specifying saturation values giving feasible trajectories. This trajectory generator features a good trade-off between computational ressources and physical constraint requirements (more complex trajectory generator could have been used as in Ref.^23^ or Ref.^24^).

### Recovery control

To achieve complete stabilization of the robot once the gap has been crossed, we implemented a recovery control procedure consisting of a control state machine inspired by Ref.^25^

The state mechanism involves four sequentially event-triggered steps depending on the conditions defined as follows:

1. Step 1: attitude stabilization: only the attitude controller is turned on with (0, 0, 0) as the required attitude on the three rotational axes (roll, pitch, and yaw) and the thrust is kept constant at $T = m.g$ to compensate for gravity. Step 2 is carried out once the following condition 1 has been reached:

\begin{align*}
{ \bf CONDITION \,1}: \left\{  { \begin{matrix} { \vert \theta \vert < 20^ \circ } \hfill \\ { \vert \phi \vert < 20^ \circ } \hfill \\ { \vert \dot \theta \vert < 10rad.{s^{ - 1}}} \hfill \\ { \vert \dot \phi \vert < 10rad.{s^{ - 1}}} \hfill \\ \end{matrix} } \right.. \tag{20}
\end{align*}

2. Step 2: vertical speed control: the feedback control of the robot's vertical speed is turned on with the required speed equal to 0. When the speed conditions defined in Equation (21) are satisfied, the required vertical position ${z_{des}}$ becomes equal to the estimated vertical position $\hat z$ just before switching to step 3.

\begin{align*}
{ \bf CONDITION \, \,2}: \begin{matrix} { \vert \dot z \vert  < 0.3m.{s^{ - 1}}} \hfill \\ \end{matrix}. \tag{21}
\end{align*}

\begin{align*}
\rightarrow {z_{des}} = \hat z.
\end{align*}

3. Step 3: Longitudinal and lateral speed control: activation of the horizontal speed control with the required speed equal to 0 and the vertical position control with a new required vertical position. When condition 3 relating to the X and Y axes [Eq. (22)] is satisfied, the position controller locks the actual estimated positions $\hat x , \hat y$ before switching to step 4.

\begin{align*}
{ \bf CONDITION \,3}: \left\{  { \begin{matrix} { \vert {v_x} \vert  <0.5 \,m.{s^{ - 1}}} \hfill \\ { \vert {v_y} \vert  <0.5 \,m.{s^{ - 1}}} \hfill \\ \end{matrix} } \right.. \tag{22}
\end{align*}

\begin{align*}
\rightarrow \left\{  { \begin{matrix} {{x_{des}} = \hat x} \hfill \\ {{y_{des}} = \hat y} \hfill \\ \end{matrix} } \right..
\end{align*}

4. Step 4: full control: activation of the attitude and position control system with new required positions ${ \bm{\xi} _{des}} = ( {x_{des}} , {y_{des}} , {z_{des}}{ ) ^t}$.

### Metrics and calibration

#### Metrics

**FIG. 10.**
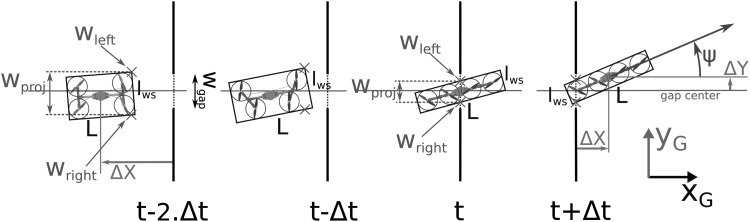
Top view during gap crossing with ${w_{left}}$ and ${w_{right}}$ and metric ${w_{proj}}$.

As the Quad-Morphing robot is the first prototype showing both morphing and gap-crossing abilities, some new metrics were adopted to show the robot's performances, especially their repeatability. The main metric adopted for this purpose was the projected wingspan on the gap plane on the Y-axis, called ${w_{proj}}$ (Fig. 10), which was defined as follows:

\begin{align*}
 { w_ { proj } } ( \gamma , \psi ) = { \frac { { l_ { ws } } ( \gamma ) }  { \cos \psi } } . \tag { 23 } 
\end{align*}

When the robot is folded, with Equation (12):

\begin{align*}
\begin{matrix} L \hfill & { = L ( \gamma = 90^ \circ ) = {l_b} + {l_a} + 2.{r_{{p_{real}}}}} \hfill \\ {{l_{ws}}} \hfill & { = {l_{ws}} ( \gamma = 90^ \circ ) = 2.{r_{{p_{real}}}}} \hfill \\ \end{matrix} , \tag{24}
\end{align*}

which leads us to avoid colliding with the gap, with the width of which is denoted as ${w_{gap}}$:

\begin{align*}
 { w_ { proj } } ( \psi ) = { \frac { 2. { r_ { { p_ { real } } } } }  { \cos \psi } } < { w_ { gap } } . \tag { 25 } 
\end{align*}

Likewise on the Z-axis in relation to the robot's height *h* and the elevation angle $\varepsilon$, which corresponds to the angle between *x_R_*, the robot's axes and *x_G_*, the gap's axes on the $( O{x_G}{z_G} )$ plane, with the height of the gap defined by ${h_{gap}}$:

\begin{align*}
 { h_ { proj } } ( \varepsilon ) = \frac { h }  { { \cos \varepsilon } } < { h_ { gap } } \tag { 26 } 
\end{align*}

with

\begin{align*}
\varepsilon = - \mathop { \tan } \nolimits^ { - 1 } \left( { { \frac { \tan \theta }  { \cos \psi } } } \right). \tag { 27 } 
\end{align*}

As shown in Figure 10, we introduced the two points denoted ${w_{left}}$ and ${w_{right}}$, which correspond to the left and right edges of the cross section between the gap and the robot's span on the Y-axis (${h_{up}}$ and ${h_{down}}$ for the top and bottom edges, respectively, on the Z-axis):

\begin{align*}
\begin{matrix} { { w_ { left } } } \hfill & { = \Delta Y + \frac
{ { { w_ { proj } } } }  { 2 } - \Delta X. \tan \psi } \hfill \\ {
{ w_ { right } } } \hfill & { = \Delta Y - \frac { { { w_ { proj }
} } }  { 2 } - \Delta X. \tan \psi } \hfill \\ { { h_ { up } } }
\hfill & { = \Delta Z + \frac { { { h_ { proj } } } }  { 2 } -
\Delta X. \tan \varepsilon } \hfill \\ { { h_ { down } } } \hfill
& { = \Delta Z - \frac { { { h_ { proj } } } }  { 2 } - \Delta X.
\tan \varepsilon } \hfill \\ \end{matrix}. \tag { 28 }
\end{align*}

Equation (28) shows the importance of having an efficient and accurate $\psi$-angle control, but this can also be said in the case of the Y-axis and Z-axis control to minimize $\Delta Y$ and $\Delta Z$, that is, the distance between the robot's center of mass and the geometrical center of the gap on the Y-axis and the Z-axis in the gap's frame ${ \cal G}$, as can be seen in Figure 10. We also considered the case where the robot is outside the gap: this situation requires new definitions of the various points of interest, as follows (Fig. 10):

**Figure:**
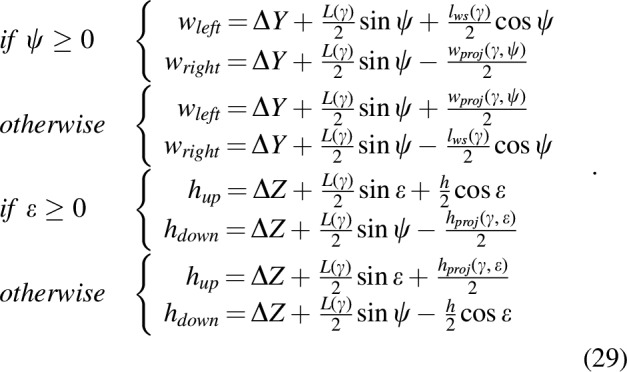


#### Calibration

To improve the robot's performances, several experiments were conducted with the same gap-crossing speed ${V_{{A_X}}} = 2.5 \,m.{s^{ - 1}}$ to compensate for constant perturbations on the Y-axis and the Z-axis during folding by biasing ($\Delta {Y_{bias}}$ and $\Delta {Z_{bias}}$) the robot's position with respect to the center of the gap. The biases were determined by finding the middle of the most extreme trajectories during our experiments when the robot's center of mass was on the same plane as the gap:

\begin{align*}
 { \rm { for } } \, \Delta X = 0: \begin{cases} \begin{matrix}
{ \Delta { Y_ { bias } } = \frac { { max ( { w_ { left } } ) + min
( { w_ { right } } ) } }  { 2 } } \\ { \Delta { Z_ { bias } } =
\frac { { max ( { w_ { up } } ) + min ( { w_ { down } } ) } }  { 2
} } \\ \end{matrix} \end{cases} . \tag { 30 }
\end{align*}

Therefore, to compensate for these biases, the required positions in the gap frame ${ \cal G}$ during the approach now become

\begin{align*}
\begin{matrix} {{Y_{des}} = - \Delta {Y_{bias}}} \\ {{Z_{des}} = - \Delta {Z_{bias}}} \\ \end{matrix}. \tag{31}
\end{align*}

## Results

### Experimental setup

The Vicon motion system was used instead of an embedded magnetometer and to determine the robot's position in real time. Our RT-MaG custom toolbox^19^ was used to run the Simulink model on our embedded Linux computer module and to monitor the data in real time with Quanser^®^ Real-Time Control (QUARC) running on the ground station. A video of the experimental setup is provided in the attached files.

### Crossing the gap

In the eight experiments presented here, we can see the robot's extreme positions during the final approach and the crossing of the gap ($20 \times 20c{m^2}$). All the positions are given in the gap frame ${ \cal G}$, where the origin is taken to be the center of the gap.

**FIG. 11.**
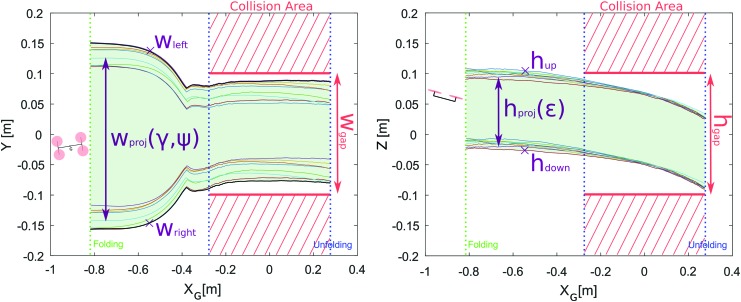
Projected wingspan and height versus relative robot position to the gap. *Green dashed lines* correspond to the position when the robot begins the folding procedure. *Blue dashed lines* correspond to the extremal positions when the robot could collide with the gap. Color images available online at www.liebertpub.com/soro

As shown in Fig. 11, the robot was able to perform eight consecutive gap-crossing trials without colliding or touching the sides of the gap. Figure 11 shows edges ${w_{left}}$ and ${w_{right}}$ (as defined in Fig. 10) in the case of each experiment versus the robot's position on the *X_G_*-axis and ${h_{up}}$ and ${h_{down}}$ give the positions of the edges of the robot on the $( O{x_G}{z_G} )$ plane. The area between the two blue dashed vertical lines indicates possible positions where the robot is liable to collide with the gap (the gap's width and height are presented in horizontal solid red lines in the figures) and the X-axis corresponds to the robot's center of mass on the *X_G_*-axis. In Figure 11, we can see the occurrence of a loss of altitude due to the lower available thrust in the folded position. Once the robot's arms have been folded, the two rotors placed just above the robot's body lead to an overlapping effect that is responsible for the loss of propeller lift. The resulting loss of altitude is not totally compensated for by the controller, which is subjected to thrust limitations to prevent the motors from being saturated and to keep full control of the robot's pitch and yaw, and thus to keep a robust control on the $( O{x_G}{y_G} )$ plane as shown in Figure 11. The thrust is limited by the propellers' intrinsic power, which did not suffice to maintain a constant flight altitude when substantial yaw perturbation occurred.

#### Attitude perturbations

**FIG. 12.**
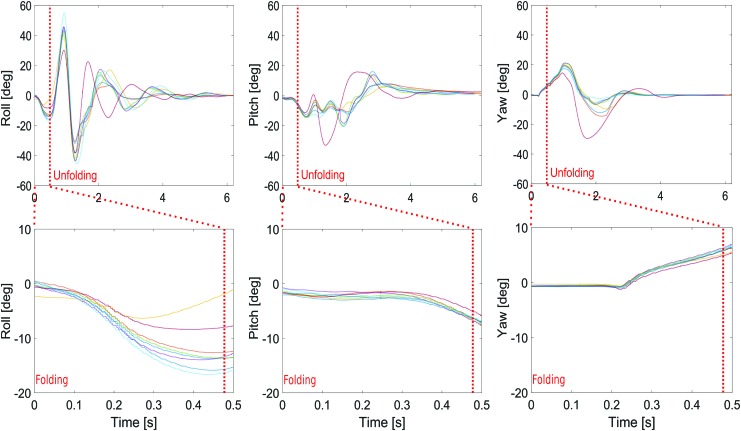
Euler angles during gap crossing and recovery procedure. Time origin corresponds to the beginning of the folding process. *Bottom row* corresponds to a zoom of the *top row*. *Red dashed lines* correspond to the beginning of the unfolding procedure. Color images available online at www.liebertpub.com/soro

In Figure 12 giving Euler angles versus time, we can see that the perturbations encountered are <10° on the controllable axis, namely the pitch and yaw axes. These perturbations were not completely rejected because of the lack of thrust power due to the inertia and the weight of the robot in the case of the present platform. It is worth noting that the folding process also induces roll perturbations of about 15°. Attitude perturbations can be explained by inertial effects due to the two arms' acceleration, which did not entirely cancel each other out during the folding process because the arm rotations were not strictly synchronized.

**FIG. 13.**
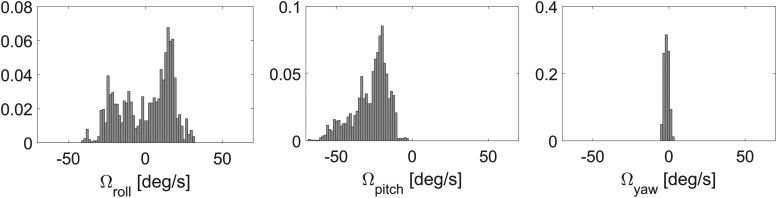
Normalized angular rate distribution before folding in the case of eight trajectories.

Figure 13 gives data on the angular rates recorded when the robot's position was 2 m ahead of the gap and when the robot started folding its wings. As expected in terms of the angular rates, this figure shows that low values (never exceeding 100°/*s*) occurred on all the axes before crossing the gap plane.

#### Comparative results

**FIG. 14.**
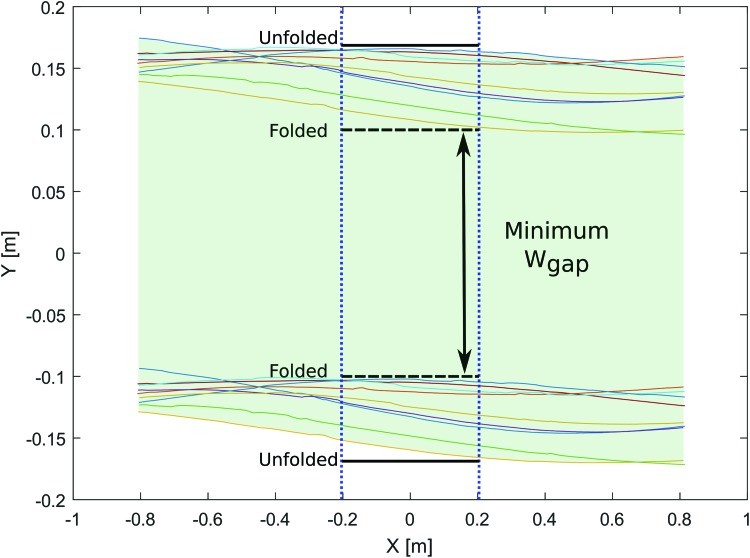
Projected wingspan with unfolded robot versus the robot's position relative to the gap. Two virtual gaps are shown with both configurations: unfolded and folded. *Blue dashed lines* correspond to the extremal positions when the robot could collide with the gap. Color images available online at www.liebertpub.com/soro

To make quantitative comparisons, we recorded nine unfolded robot flights without the gap at the same speed (${V_X} = 2.5 \,m.{s^{ - 1}}$) and recorded the results presented in Table 1 and Figure 14. These figures give an idea of the robot's performances on lateral axis Y in comparison with the folded robot scenario: it can be seen here that the robot can pass safely through a gap that has a minimum width of ${ ( {w_{gap}} ) _{min}} = 332 \,mm$. This result can be compared with the minimum gap width ${ ( {w_{gap}} ) _{min}} = 180 \,mm$ with the folded robot. Minimum passable gaps are plotted in black in Figure 14.

**Table 1.** Results Obtained With and Without Morphing Abilities

	${ \left( {{w_{proj}}} \right) _{max}}$	${ \left( { { \frac { { w_ { proj } } } { { l_ { ws } } } } } \right) _ { max } }$	${ \left( {{w_{gap}}} \right) _{min}}$
With morphing abilities	134 mm	105%	180 mm
Without morphing abilities	268 mm	100%	332 mm

The maximum projected wingspan ${ ( {w_{proj}} ) _{max}}$ observed during all these experiments was also compared with the robot's wingspan while crossing the gap ${ ( { \frac { { w_ { proj } } }  { { l_ { ws } } } } ) _ { max } }$. The projected wingspan value (105% of the actual wingspan value) in the morphing robot can be explained by the yaw perturbations that occurred during the folding process.

We, therefore, sought to determine the minimum passable gap width in the case of each configuration (with/without morphing abilities). To quantify the advantages of this novel configuration, we took the ratio between the gap size that could be crossed by the robot with and without morphing abilities:

\begin{align*}
 { \eta _ { meas } } = { \frac { { { ( { w_ { gap } } ) } _ { min , { \rm { with } } \, { \rm { morph } } } } }  { { { ( { w_ { gap } } ) } _ { min , { \rm { without } } \, { \rm { morph } } } } } } = 54 \%. \tag { 32 } 
\end{align*}

This value can be compared with $\eta = 48 \%$, which is the theoretical gain in the passable gap width in the case of the morphing robot (as detailed in Modeling the folding system section).

## Conclusion

In this article, a new aerial robotic platform endowed with morphing capabilities is presented. The control laws developed for our Quad-Morphing robot were based on a large body of literature on the nonlinear control of quadrotors, including aspects such as attitude estimation and trajectory planning. As observed in studies on birds, our robot can suddenly reduce its wingspan by 50% in a very short time (about 250 ms) to pass through a gap such as a small square aperture that can be equal to 54% the robot's unfolded wingspan. We have also presented a gap-crossing scenario with a crossing speed as fast as $2.5 \,m.{s^{ - 1}}$, which is similar to the forward speed of birds in flight.^26^

As bird's wings tuck back against their body, the particularity of our morphing robot is its ability to reduce its wingspan, resulting in an inevitable loss of roll control due to the alignment of the four rotors when the robot is in its folded state. During aperture crossing, maximum roll perturbations of up to 15° can occur, but these could be reduced in the future if we can find a means of preserving the robot's roll control when it is in the folded configuration, by tilting the rotors, for example, as proposed by Ref.^8^

By adopting new metrics for characterizing the performances of the morphing robot, we have shown that adjusting the yaw angle (<7° here) directly affects the robot's gap-crossing performances (by dramatically increasing the projected wingspan). Better performances could no doubt be achieved by improving the thrust-to-weight ratio (1.5 in the case of our platform) and the rotor's drag coefficient, or by introducing tilting rotors giving yaw control with thrust.

This novel morphing structure does not have to make aggressive maneuvers to make the aerial robot cross a gap at high speed. Morphing improves the trade-off between payload and fast dynamics. The same morphing principle could be applied to a bigger and heavier quadrotor, assuming that the rotor folding mechanism shows sufficiently fast dynamics. Without having to reduce the payload, it should be possible to equip a future morphing quadrotor with a relatively bulky fixed camera so that autonomous gap crossing can be based entirely on an embedded visual system. Unlike strategies requiring aggressive maneuvers, the angular rates of the Quad-Morphing robot's body consistently reached low values here (always <100°/*s*) during the approach phase toward the gap, which can be easily compensated for when implementing an accurate vision-based positioning system. When a robot is flying toward a gap, taking the decision to fold up its structure or not on the basis of visual cues alone will be the next challenge to be met by the Quad-Morphing robot.

## Supplementary Material

Supplemental data
